# The attenuation effect of low piperine *Piper nigrum* extract on doxorubicin-induced toxicity of blood chemical and immunological properties in mammary tumour rats

**DOI:** 10.1080/13880209.2021.2018470

**Published:** 2021-12-28

**Authors:** Jirakrit Saetang, Aman Tedasen, Surasak Sangkhathat, Natnaree Sangkaew, Sirinapa Dokduang, Napat Prompat, Siriporn Taraporn, Potchanapond Graidist

**Affiliations:** a Department of Biomedical Sciences and Biomedical Engineering, Faculty of Medicine, Prince of Songkla University, Hat Yai, Thailand; b Department of Surgery, Faculty of Medicine, Prince of Songkla University, Hat Yai, Thailand; c EZ-Mol-Design Laboratory, Faculty of Medicine, Prince of Songkla University, Hat Yai, Thailand; d Department of Medical Technology, School of Allied Health Sciences and Public Health, Walailak University, Thai Buri, Thailand

**Keywords:** PFPE, cytokine, Th1, Th2, Treg

## Abstract

**Context:**

Many natural extracts have been shown to minimize the toxicity of doxorubicin (Dox). Low piperine *Piper nigrum* L. (Piperaceae) extract (PFPE) is a natural extract containing many types of antioxidants that may reduce Dox toxicities.

**Objective:**

To evaluate the effect of PFPE in attenuating the side effects of Dox.

**Materials and methods:**

Tumour-bearing Sprague Dawley rats were divided into five groups including normal, vehicle, 100 mg/kg BW of PFPE plus 2 mg/kg BW of Dox (P100 + Dox), 100 mg/kg BW of PFPE plus 2 mg/kg BW of Dox (P200 + Dox) and Dox. Rats were treated with Dox and/or PFPE three times/week for 4 weeks. Tumour burden, blood parameters, weight of internal organs and immunological data were investigated.

**Results:**

The addition of 200 mg/kg PFPE significantly restored the levels of AST from 174.60 ± 45.67 U/L in the Dox group near to normal levels at 109.80 ± 4.99 U/L. The combination of PFPE and Dox also decreased the levels of CXCL7, TIMP-1, sICAM-1 and l-selectin about 1.4–1.6-fold compared to Dox group. Feeding rats with 200 mg/kg BW of PFPE combination with Dox slightly increased Th1 from 161.67 ± 14.28 cells in Dox group to 200.75 ± 5.8 cells meanwhile suppressed Treg from 3088 ± 78 cells in Dox to 2561 ± 71 cells.

**Discussion and conclusions:**

This study showed that PFPE ameliorated Dox toxicity in many aspects indicating the role of antioxidant and other substances in the extract on toxicity attenuation. This suggested the using of PFPE may be valuable for Dox treated patients.

## Introduction

Breast cancer is the most common site-specific cancer and the leading cause of cancer-related death in women worldwide (Alkabban and Ferguson 2020). For global cancer incidence in 2020, breast cancer is estimated at 2.3 million new cases and represented at 11.7% of all cancer cases. Transitioned countries have higher incidence rates of breast cancer more than transitioning countries. The highest incidence rates were in Australia/New Zealand followed by Western Europe, Northern America and Northern Europe. Meanwhile, the lowest rates are in Central America, Eastern and Middle Africa, and South Central Asia. Nevertheless, women living in transitioning countries have higher mortality rates than transitioned countries (Sung et al. 2021).

In clinical practice, there are two usual treatments for breast cancer: surgery for local control and chemotherapy and/or hormonal therapy for preventing metastatic relapse (Alkabban and Ferguson 2020). Among other chemotherapeutic agents, doxorubicin (Dox) has been generally applied in the treatment of breast cancer patients who are resistant to hormonal therapy, or with metastasis (Gariboldi et al. 2003). The well-known mechanism of action is the intercalating properties on double-stranded DNA, leading to free radical formation and oxidative stress, which then cause tumour cell death (Ackland et al. 2001). Although this chemotherapeutic agent is one of the most effective drugs in breast cancer treatment, many adverse effects have been reported in clinical practice, especially hematotoxicity, nausea and vomiting, hair loss (Baltali et al. 1999), irreversible cardiomyopathy (Chatterjee et al. 2010) and accelerated bone loss (Rana et al. 2013). Moreover, immunological changes have also been demonstrated in breast cancer patients receiving chemotherapeutic drugs since the depletion of leukocytes and bone marrow damage (Nurgalieva et al. 2011) were found in breast cancer patients after chemotherapy (Wijayahadi et al. 2007; Verma et al. 2016), making them more sensitive to infections (Wise 2016).

Using natural compounds as a supplement or adjuvant for treating cancer patients is very attractive nowadays. In addition to a wide variety of effects against cancer, the evidence of natural compounds' ability to minimize the toxicity from chemotherapeutic regimens is growing in standard cancer therapies (Serna-Thomé et al. 2018). For example, *Lentinula edodes* (Berk) Pegler (Omphalotaceae) mycelia extract (LEM) prevented a chemotherapy-induced decrease in the number of activated NK and NKT cells (Nagashima et al. 2013). Proanthocyanidins extracted from grape seeds were also reported to have the ability to attenuate myocardial oxidative stress, promote NK cells cytotoxicity, induce IFN-γ expression and enhance lymphocyte proliferation in Dox treated tumour-bearing mice (Zhang et al. 2005). Moreover, the ethanol extract of *Picria fel-terrae* Lour. (Scrophulariaceae) seemed to reduce Dox-mediated immune suppression in rats by promoting CD4^+^ and CD8^+^ T cells (Lubis et al. 2019). The same results were also found in rats co-treated with Dox and *Myrmecodia tuberosa* Jack (Rubiaceae) hypocotyl extract (Sumardi et al. 2013).

Black pepper, or *Piper nigrum* L. (Piperaceae), is an important medicinal plant with a long history of use in the compositions of traditional medicines in numerous tropical countries. The extract of *P. nigrum* consists of various alkaloids, terpenes, steroids, lignans, flavones and alkamides, which contribute to antioxidant, antibacterial, antifungal and anticancer activities (Butt et al. 2013). However, the major substance in the black pepper extract, piperine, has been reported for its toxicity through the induction of reactive oxygen species and hydroxyl radicals in rat testes (Daware et al. 2000; Chinta et al. 2017). Another study revealed that piperine promoted the stability of *Aspergillus* derived aflatoxin B1 in the liver (Allameh et al. 1992). To avoid these unsatisfactory effects, we improved this extract by eliminating piperine from the black pepper extract using a specific method (Sriwiriyajan et al. 2014). Interestingly, this extract demonstrated a strong antitumor effect in both the preventive and therapeutic aspects of mammary tumour-bearing rats and cholangiocarcinoma cell lines (Sriwiriyajan et al. 2014, 2016; Tedasen et al. 2020). Importantly, the fraction of *P. nigrum* with low levels of piperine showed more toxicity against several breast cancer cell lines than piperine containing fractions (Sriwiriyajan et al. 2014). In addition to the direct apoptosis inducing effect on tumours, PFPE also promoted antitumor immunity by regulating Th1/Th2/Treg cells (Saetang et al. 2021).

Although the cancer inhibitory mechanism of PFPE was demonstrated with a low toxicity, the result of PFPE treatment on chemotherapy-induced adverse events is still unclear. Since many reports have shown some antioxidants have immunomodulatory function which improved the immune response in animal models or increased the quality of life of anthracycline treated breast cancer patients (Zhang et al. 2005; Nagashima et al. 2013, 2017; Sergazy et al. 2020), we tried to evaluate the effect of PFPE, which consisted of a variety of antioxidants and lowered the toxicity of piperine, in Dox-induced adverse events. This included the haematological and immunological parameters, the specific immune cells associated with antitumor immunity, including Th1, Th2 and Treg cells, and cytokine array analysis.

## Materials and methods

### Chemicals

All chemicals including dichloromethane, diethylether and butylated hydroxytoluene used were of analytical grade from Sigma-Aldrich (St. Louis, MO). Doxorubicin (2 mg/mL) was purchased in solution form (ADRIM, New Delhi, India) and stored at 4 °C. *N*-methylnitrosourea (NMU) (Sigma-Aldrich, St. Louis, MO, catalogue no. N4766) was prepared at the concentration of 100 mg/mL by diluting it with 0.9% normal saline solution pH 4.0–5.0. Estradiol (Sigma-Aldrich, St. Louis, MO, catalogue no. E8875) was prepared in absolute ethanol (Merck, Kenilworth, NJ) at the concentration of 100 mg/mL. On the day of rat feeding, Estradiol was diluted in sesame oil (food grade) before administration at the concentration of 100 μg/mL.

### Preparation of PFPE

The black peppercorn (*P. nigrum*) was collected from Nakhon Si Thammarat Province in Thailand with the voucher specimen number SKP 146161401, identified by Assistant Professor Supreeya Yuenyongsawad and deposited in the herbarium at the Southern Centre of Thai Traditional Medicine, Department of Pharmacognosy and Pharmaceutical Botany, Prince of Songkla University, Hat Yai, Thailand. PFPE extract was prepared according to the previous work (Sriwiriyajan et al. 2016). Briefly, dry powder of *P. nigrum* fruit was prepared and soaked in dichloromethane, and then concentrated with a rotary evaporator (BUCHI, Flawil, Switzerland) at 45 °C. Piperine was then crystallized by the addition of cold diethyl ether and separated using Whatman filter paper (Whatman Bioscience, Cambridge, UK). Finally, the extract was concentrated by an evaporator at 45 °C and kept at room temperature until use. The composition and biological activity of PFPE were confirmed using gas chromatograph-mass spectrometer (GC–MS; Hewlett Packard, Houston, TX) and MTT assay (against breast cancer cells, MCF-7) (Tedasen et al. 2020) (Figure S2).

### Mammary tumour induction and treatment

Female Sprague Dawley rats, 45 days old (virgin) and weighing between 150 and 180 g, were used in this study. All rats were purchased from Nomura Siam International Co., Ltd. (Bangkok, Thailand), and maintained in the Southern Laboratory Animal Facility, Faculty of Science, Prince of Songkla University, Hat Yai, Thailand. The conditions of housing were: temperature of 22 ± 3 °C, relative humidity of 30 ± 10% and 12 h light–dark cycle. The experiment was conducted under guidelines approved by the Animal Care and Use Committee of Prince of Songkla University (animal experimentation application ref. 44/2019).

For tumour induction, the protocol was modified from Rivera et al. (1994) and our previous work (Tedasen et al. 2020). Briefly, 50-day-old rats were injected intraperitoneally with 250 mg/kg BW of NMU, followed by another round of injections at 80 days old. After seven days of NMU injections, rats were orally administered 100 μg/kg BW of Estradiol for 10 days.

For treatment study, rats were divided into five groups, including normal (negative control), vehicle (negative control), 100 mg/kg BW of PFPE combination with 2 mg/kg BW of Dox (P100 + Dox), 200 mg/kg BW of PFPE combination with 2 mg/kg BW of Dox (P200 + Dox) and Dox (Dox). The dose of Dox obtained from the preliminary experiment demonstrated 2 mg/kg BW i.p. three times a week for 4 weeks to a total of 24 mg/kg (similar to a dose of 150 mg/m^2^ for humans) was the response dose for the NMU-induced mammary tumour model (data not shown). PFPE treatment doses were also from the previous results for treating rats in this model (Deng et al. 2016; Sriwiriyajan et al. 2016). The normal group received no induction and treatment; meanwhile, the other groups were induced with *N*-nitrosomethylurea (NMU) for tumour formation. The treatments were started when the first tumour showed a diameter of 0.2 mm. All rats received their specific treatment three times a week for 4 weeks. The schedule was every Monday, Wednesday and Friday of each week. PFPE and vehicle were administered orally while Dox was injected intraperitoneally. The tumour size was measured horizontally and vertically using a Vernier calliper, and calculated with the formula *V* = 0.4*ab*^2^, where *a* and *b* represented the largest tumour diameter and the next largest tumour diameter, respectively (Tedasen et al. 2020). At the end of the experimental period, rats were anaesthetized and sacrificed. Blood was collected by cardiac puncture for haematologic and biochemical analysis. The serum was separated by centrifugation for cytokine array analysis. Tumours and organs (including lung, liver, kidney, heart and stomach) were observed and the weight recorded.

### Live animal imaging

IntegriSense 680 (PerkinElmer, Waltham, MA; catalogue no. NEV10645) was used to monitor the size of the tumour in each group of rats. Briefly, a representative rat was anaesthetized and intravenously injected with 12 nmol/600 μL of IntegriSense 680 at days 10 and 24 of the experimental period. The measurement of fluorescent signal was performed after 24 h of fluorescence dye injection using the IVIS Lumina III system (PerkinElmer, Waltham, MA). The average radiant efficiency obtained from the Living Image software (supplied with IVIS Lumina III system) was used to indicate the intensity of the fluorescent signal.

### Laboratory analysis

Complete blood cell count was performed on the ADVIA 120 automated haematology analyser (Bayer Diagnostics, Reading, UK) with optical laser light scattering for cell enumeration, flow cytometer and laser diffraction. Serum chemistry was analysed on an automated analyser with an attached ion-selective electrode module (Cobas Mira, Roche Diagnostic Systems, Indianapolis, IN) following the protocol from the company.

### Mononuclear cells isolation and flow cytometry

Blood was collected by cardiac puncture of the experimental rats. The density gradient centrifugation was performed to isolate peripheral blood mononuclear cells using Lymphoprep (Stemcell Technologies, Vancouver, Canada, catalogue no. 07851). The blood was mixed with serum free-RPMI 1640 medium (Gibco, Carlsbad, CA, catalogue no. 31800105) at a 1:1 ratio and then overlaid on Lymphoprep gradient medium. After centrifugation at 800×*g* for 30 min, the peripheral blood mononuclear cells were collected from the interphase between the plasma and Lymphoprep layers and counted. Mononuclear cells (2 × 10^6^ cells/mL) were then incubated with Leukocyte Activation Cocktail, with BD GolgiPlug (BD Biosciences, Franklin Lakes, NJ, catalogue no. 550583) in completed RPMI medium for 4 h at 37 °C in a CO_2_ incubator. Cells were then washed and stained with FITC mouse anti-rat CD4 (catalogue no. 554843) and/or PE mouse anti-rat CD25 (catalogue no. 554866) antibodies (BD Biosciences, Franklin Lakes, NJ). After fixation with fixation buffer (BD Biosciences, Franklin Lakes, NJ, catalogue no. 554655) for 20 min at 4 °C, cells were then washed with permeabilization wash buffer (BD Biosciences, Franklin Lakes, NJ, catalogue no. 554722). Stained cells were incubated with Alex 647 mouse anti-rat IFN-γ (BD Biosciences, Franklin Lakes, NJ, catalogue no. 562213) and/or PE mouse anti-rat IL-4 antibodies (BD Biosciences, Franklin Lakes, NJ, catalogue no. 555082) at 4 °C for 30 min in a dark environment. The stained cells were then washed and resuspended in cell staining buffer. Flow cytometry analysis was performed using the Beckman CytoFLEX S platform (Beckman Coulter, Brea, CA). The results were analysed using Kaluza analysis flow cytometry software (Beckman Coulter, Brea, CA).

### Cytokine array assay

Proteome profiler rat cytokine array kit, panel A (R&D Systems, Minneapolis, MN, catalogue no. ARY008) was used to evaluate the difference in cytokine levels in each group of rats. Each spot of the membrane was coated with 29 types of specific antibodies of rat cytokine, including CXCL1/CINC-1, IL-1ra/IL-1F3, l-selectin, CXCL3/CINC-2α/β, IL-2, CXCL9/MIG, CXCL2/CINC-3, IL-3, CCL3/MIP-1α, CNTF, IL-4, CCL20/MIP-3α, fractalkine/CXC3CL1, IL-6, CCL5/RANTES, GM-CSF, IL-10, CXCL7/thymus chemokine, ICAM-1, IL-13, TIMP-1, IFN-γ, IL-17, TNF-α, IL-1α/IL-1F1, CXCL10/IP-10, VEGF, IL-1β/IL-1F2 and LIX. According to the manufacturer’s protocol, a pooled plasma sample from five rats in each group was mixed with a detection antibody cocktail and incubated for 1 h at room temperature. During this period, the membrane was incubated with a blocking buffer for 1 h at room temperature and then soaked with a pre-mixed pooled plasma sample overnight at 4 °C. The membranes were then subsequently incubated with HRP-conjugated streptavidin for 30 min at room temperature. The signal was evaluated after exposure to peroxidase substrate using Alliance Q9 (Uvitec, Cambridge, UK). The intensity of the signal was scored using UVITEC Alliance software (Uvitec, Cambridge, UK). The data were expressed as the fold change with correspondence to the normal group.

### Statistical analysis

Data were analysed by one-way ANOVA. All data were expressed as mean ± SEM. Multiple conditions were analysed using a parametric one-way ANOVA using SPSS, version 24.0 (IBM Corp., Armonk, NY). A *p* value <0.05 was considered statistically significant.

## Results

### GC–MS analysis revealed that more than 50 compounds were found in PFPE

After the extraction, the yield of PFPE extracted from the raw material was approximately 2.2% with a sticky, oily and black colour in appearance. GC–MS analysis of PFPE indicated the presence of more than 50 phytochemical constituents in three major groups, including, terpene, alkaloid and lignan. The first 15 major compounds constituted more than 58% of PFPE. Moreover, due to the crystallization step in the extraction protocol, piperine was found approximately 0.99% in PFPE. Among the top 15 major phytochemicals, 10 compounds were reported to have antibacterial, anticancer and anti-inflammation (Table 1).

**Table 1.** Phytochemical ranking found in PFPE using GC–MS analysis.

Ranking	Name of the compound	Molecular formula	Peak area %	MW (g/mol)	Role
1	Caryophyllene	C_15_H_24_	6.46	204.35	a
2	Limonene	C_10_H_16_	4.84	138.25	a, b, c
3	beta-Selinene	C_15_H_24_	4.75	204.35	b
4	Pellitorine	C_14_H_25_NO	4.57	223.3544	c
5	Piperlonguminine	C_16_H_19_NO_3_	4.48	273.33	c
6	1,4,7,-Cycloundecatriene	C_15_H_24_	4.03	204.35	–
7	Neo-allo-ocimene	C_10_H_16_	3.72	136.23	–
8	alpha-Selinene	C_15_H_24_	3.57	204.35	–
9	Pipericine	C_22_H_41_NO	3.53	335.567	–
10	beta-Elemene	C_15_H_24_	3.39	204.35	c
11	Guineensine	C_24_H_33_NO_3_	3.15	383.5237	b
12	beta-Bisabolene	C_15_H_24_	3.10	204.35	c
13	2,4,14-Eicosatrienamide	C_24_H_43_NO	2.94	361.6	–
14	Caryophyllene oxide	C_15_H_24_O	2.84	220.3505	c
15	Kusunokinin	C_21_H_22_O_6_	2.71	370.4	c

a: antioxidant; b: anti-inflammation; c: anticancer.

### PFPE did not disrupt the anticancer properties of doxorubicin

**Figure 1.**
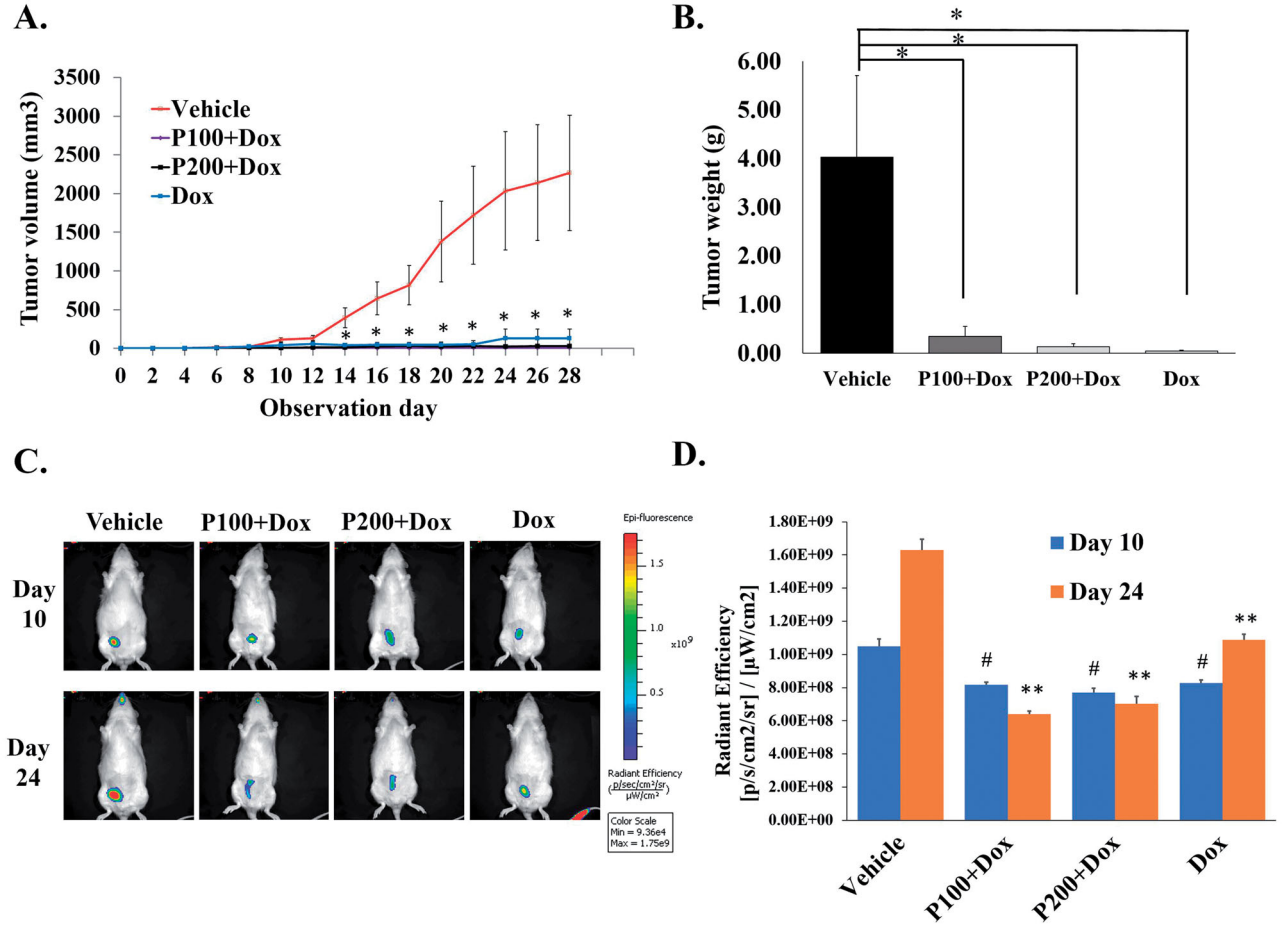
Anticancer effects of doxorubicin and doxorubicin combination with PFPE in NMU-induced mammary tumour rats. After tumour induction, rats were treated with vehicle (5% medium chain fatty acid and 2% vitamin E), 2.0 mg/kg of doxorubicin, and 2.0 mg/kg BW of doxorubicin combination with 100 or 200 mg/kg BW of PFPE. (A) Tumour size was recorded during the treatment period. (B) At the end of the study, tumours were isolated and weighted. (C) Imaging was performed 24 h after IntegriSense 680 injection. Breast tumour presented at the breast position on the right hind leg of the rats. (D) The formed tumour was quantified by measuring radiant efficacy. Data represent the mean ± SEM (*n* = 5). *p < 0.05, compared to vehicle group. For (D), ^#^*p* < 0.05, compared to vehicle group at day 10; **p < 0.05, compared to vehicle group at day 24. One-way ANOVA was used for all analyses.

In order to determine the antitumor suppressing activity of Dox and Dox combination with PFPE, mammary tumour bearing rats were treated with Dox with or without 100 or 200 mg/kg BW of PFPE. The treatment was processed three times/week for 4 weeks with the monitoring of tumour volume. The results showed that the tumours in Dox and combination of Dox with PFPE treated groups exhibited very slow progression while aggressive tumour growth was found in the vehicle group (Figure 1(A)). No significant difference in tumour volume was observed between P100 + Dox, P200 + Dox and the Dox treated groups (Figure 1(A)). On the sacrifice day, the tumours from each group of rats were isolated and weighed. Rats receiving P100 + Dox, P200 + Dox and Dox showed statistically lower tumour weights than the tumours in the vehicle group, around 323-, 75- and 17-fold, respectively (*p* < 0.001 for all). However, no significant difference between these three Dox treated groups was observed (Figure 1(B)). In addition, the tumour size was evaluated by specific fluorescence conjugated probe (IntegriSense 680) at 10 and 24 days after treatment. All groups of rats showed a slight difference in tumour size at 10 days, meanwhile, the dramatic changes were found in P100 + Dox, P200 + Dox and Dox treatments at 24 days compared to the vehicle group (Figure 1(C)). The signal intensity also displayed the same result as *in vivo* imaging. The average radiant efficiency of the tumours in P100 + Dox, P200 + Dox and Dox groups was between 7.71 and 8.29 × 10^8^ (p/s/cm^2^/sr)/(µW/cm^2^) in range, which was significantly lower than the vehicle group by approximately 1.3-fold (10.5 × 10^8^ (p/s/cm^2^/sr)/(µW/cm^2^)) (Figure 1(D)). Therefore, these results indicate that the combination of Dox with PFPE could suppress tumour growth and PFPE was not affected by the tumour inhibitory properties of Dox.

### PFPE reduced the side effects of doxorubicin on the weight of the internal organs of rat

**Figure 2.**
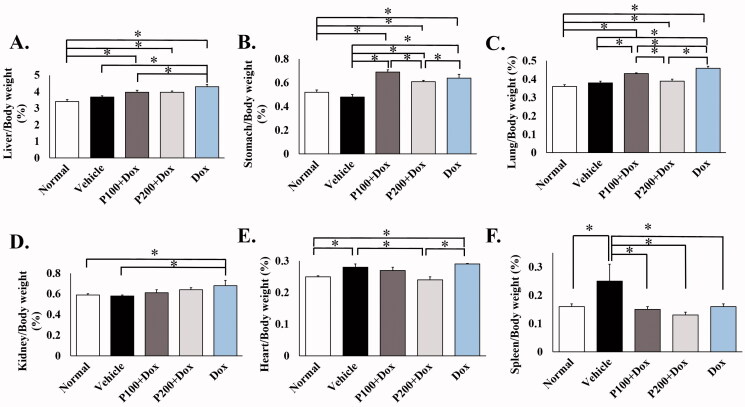
Effect of doxorubicin and the combination of PFPE and doxorubicin on organ/body weight ratio. At the termination day, rats in all groups were sacrificed and organs were collected including (A) liver (B) stomach, (C) lung, (D) kidneys, (E) heart and (F) spleen. Data represent the mean ± SEM (*n* = 5). *p < 0.05, compared as indicated using one-way ANOVA.

Many works have demonstrated the use of Dox caused toxic results in some internal organs, including the liver, kidney and heart (Tacar et al. 2013). To evaluate whether PFPE attenuates the effects of Dox, the internal organs of treated rats were observed. The results showed that all Dox treatments significantly increased the organ/body weight ratio of the liver, stomach, lung, kidney and heart by approximately 1.02–1.43-fold compared to the normal or vehicle groups. Treatment with vehicle alone increased the organ/body weight ratio of the heart and spleen. However, when 100 and 200 mg/kg BW of PFPE were included in the experiment, the organ/body weight ratios of the liver, stomach, lung and heart were statistically reduced at 1.05–1.18-fold when compared with Dox, a single treatment. Moreover, the heart showed a reduced organ/body weight ratio of about 1.16-fold in the 100 mg/kg PFPE supplement group compared to vehicle (*p* = 0.004), while the decreased organ/body weight ratio of the spleen was found to be approximately 1.56 times for 100 mg/kg PFPE (*p* = 0.016) and 1.92 times for 200 mg/kg PFPE supplement (*p* = 0.004), compared to vehicle. Interestingly, PFPE treatment, especially at the combination of Dox and 200 mg/kg BW of PFPE, brought the organ/body weight ratios of the heart and kidney back to the normal level (Figure 2). These results suggest that PFPE could reduce the toxicity of the chemotherapeutic drug, Dox.

### PFPE restored the normal levels of AST, cholesterol and triglyceride

To confirm the effect of PFPE as a chemopreventive agent, the clinical chemistry of blood samples was used as an indicator for the adverse events of rats treated with the chemotherapeutic drug, Dox. Results showed that the blood urea nitrogen (BUN), creatinine, albumin, AST, ALT, alkaline phosphatase, cholesterol and triglyceride values of the Dox treatment group were significantly changed when compared with the vehicle and normal groups. Meanwhile, creatinine and AST were restored to the normal line in Dox combination with 100 and mg/kg BW of PFPE. Surprisingly, AST, cholesterol and triglyceride were obviously reduced to a normal value (96.6 ± 14.94 U/L) when the rats were treated with Dox combination with 200 mg/kg BW of PFPE compared to the Dox group.

The synergistic effect of Dox and PFPE was found in BUN. We found that P100 + Dox suppressed the levels of BUN about 1.73-fold when compared to normal rats and 1.15 times compared to the Dox group. The dose of 200 mg/kg of PFPE plus Dox also decreased the levels of BUN approximately 2.42- and 1.62-fold compared to the normal and Dox groups, respectively. No change of bilirubin was observed in the normal, vehicle and all treatment regimens. The combination of Dox with PFPE did not help to restore alkaline phosphatase (Table 2). These results revealed that PFPE could reduce the side effects of Dox.

**Table 2.** Clinical chemistry values of the study on the induced mammary tumorigenesis in rats.

Clinical chemistry values	Normal	Vehicle	P100 + Dox	P200 + Dox	Dox
BUN (mg/dL)	28.2 ± 1.15^b,c,d,e^	21.68 ± 0.62^a,c,d,e^	16.34 ± 0.86^a,b,d,e^	11.66 ± 0.34^a,b,c,e^	18.84 ± 0.33^a,b,c,d^
Creatinine (mg/dL)	0.45 ± 0.01^b,d^	0.53 ± 0.02^a,c,e^	0.46 ± 0.01^b.d^	0.54 ± 0.02^a,c,e^	0.41 ± 0.03^b,d^
Albumin (g/dL)	4.07 ± 0.08^b^	4.29 ± 0.06^a,c,d,e^	3.00 ± 0.09^b,d^	3.44 ± 0.07^b,c,e^	3.08 ± 0.22^b,d^
Total bilirubin (mg/dL)	0.06 ± 0.02	0.06 ± 0.01	0.04 ± 0.01	0.05 ± 0.01	0.05 ± 0.01
AST (U/L)	96.60 ± 14.94^e^	126.40 ± 23.23	111.00 ± 7.21	109.80 ± 4.99	174.60 ± 45.67^a^
ALT (U/L)	38.40 ± 3.85^d,e^	40.50 ± 0.96^c,d,e^	32.60 ± 0.93^b,d^	24.60 ± 2.38^a,b,c,e^	31.60 ± 1.29^a,b,d^
Alkaline phosphatase (U/L)	73.60 ± 4.03^c,d,e^	75.20 ± 5.17^c,d,e^	50.8 ± 2.65^a,b^	53.20 ± 10.31^a,b^	41.80 ± 1.69^a,b^
Cholesterol (mg/dL)	92.80 ± 5.93^c,e^	85.20 ± 4.12^c,e^	137.20 ± 12.03^a,b,d^	86.80 ± 6.70^c,e^	124.25 ± 15.80^a,b,d^
Triglycerides (mg/dL)	185 ± 29.30^c,e^	141.60 ± 29.04^c,e^	354.20 ± 58.80^a,b,d^	90.60 ± 19.55^c,e^	326.80 ± 86.70^a,b,d^

AST: aspartate aminotransferase; ALT: alanine aminotransferase; BUN: blood urea nitrogen.

Values represented the mean ± SEM. *p* < 0.05 significantly different compared with the a = vs. normal, b = vs. vehicle, c = vs. P100 + Dox, d = vs. P200 + Dox and e = vs. Dox.

### PEPF effect on the cytokine pattern during doxorubicin treatment

**Figure 3.**
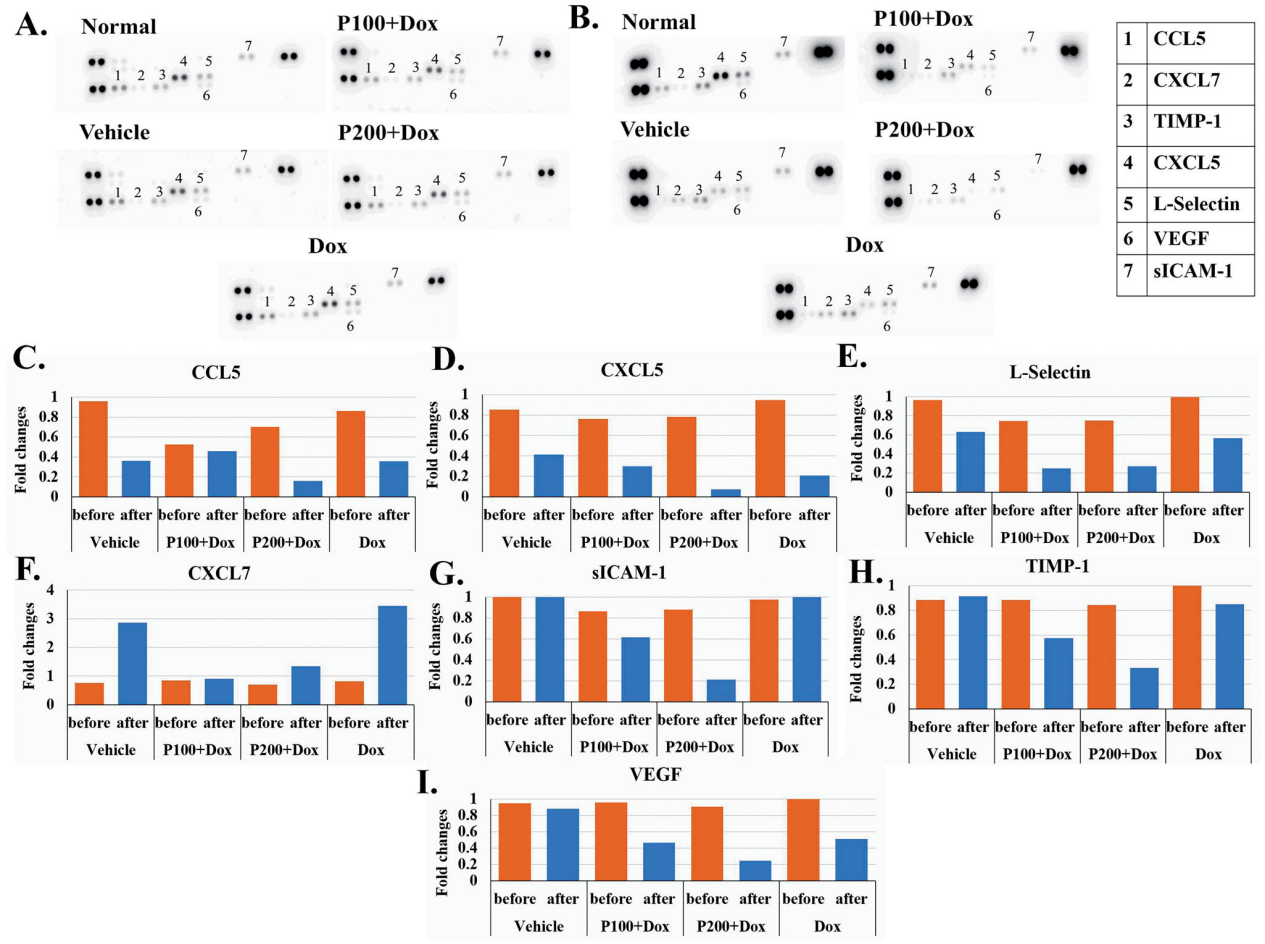
Cytokine array analysis of mammary tumour bearing rats treated with different regimens of doxorubicin and PFPE. The signal intensity of different cytokine from each group of rats is shown as a chemiluminescent spot at (A) before NMU injection and (B) sacrificed day. The signal intensity of (C) CCL5, (D) CXCL7, (E) TIMP-1, (F) CXCL5, (G) sICAM-1, (H) l-selectin and (I) VEGF was analysed and represented as fold-change compared to normal rats at the same day of blood collection. The control spots were displayed as signal without number.

To study the effect of PFPE on cytokines during chemotherapy treatment, the pooled plasma of each treatment group was used to determine cytokine patterns associated with breast cancer. The chemiluminescent intensity signal of each cytokine of each treatment was displayed as fold-change responding to the level of normal rats at before NMU injection and after treatment. In this study, seven cytokines including CCL5, CXCL7, TIMP-1, CXCL5, sICAM-1, l-selectin and VEGF were found. Results showed that, at the end of the study, three cytokines (CCL5, CXCL5 and l-selectin) were decreased in all the groups of rats. Moreover, one cytokine (CXCL7) was increased at 3.78-fold in Dox and 4.26-fold in the vehicle groups compared to the day before tumour induction. Interestingly, combining PFPE in the Dox treatment group obviously reduced the cytokine levels on the last day. Six cytokines (CCL5, TIMP-1, CXCL5, sICAM-1, l-selectin and VEGF) of both combination treatments, especially 200 mg/kg BW of PFPE, showed lower levels than treatment with Dox alone on the sacrifice day (Figure 3). These results suggest that PFPE suppressed cancer related cytokines.

### PFPE restored the normal ranges of red blood cells, white blood cells, lymphocytes and monocytes during doxorubicin treatment

Since the immunological toxicity of Dox has been recognized, we tried to determine whether PFPE alleviates the side effects of Dox on the systemic immune response of mammary tumour rats. The haematologic values including red blood cells, platelets, white blood cells, lymphocytes, neutrophils, monocytes, basophils and eosinophils of each group of treatment were determined on sacrifice day. The results showed that the increasing levels of neutrophils and eosinophils were found in the vehicle group for 1.45- and 2.00-fold, respectively. Interestingly, the opposite trend of response of eosinophils was noticeable after Dox treatment compared to the vehicle group. Moreover, when compared to the normal group, Dox treatment significantly suppressed the levels of red blood cells (1.64-fold; *p* < 0.001), white blood cells (1.40-fold; *p* = 0.009), lymphocytes (2.10-fold; *p* < 0.001) and eosinophils (2.00-fold; *p* = 0.032), whereas the number of neutrophils, monocytes and basophils was statistically increased in the Dox group at approximately 2.17-, 2.44- and 23-fold, respectively, compared to normal rats. Interestingly, when tumour bearing rats were fed PFPE, the number of red blood cells, lymphocytes, neutrophils and eosinophils was slightly restored to near normal levels. Moreover, PFPE at dose 200 mg/kg BW increased the number of white blood cells at 1.93-fold (*p* < 0.001) compared to Dox treatment. However, this dose of PFPE suppressed the levels of monocytes at approximately 3.76-fold (*p* < 0.001) compared to the Dox group. Nonetheless, Dox combination with PFPE at doses of 100 and 200 mg/kg BW increased the levels of platelets, which was not found in any group of rats (Table 3).

**Table 3.** Haematological values of the study on the induced mammary tumorigenesis in rats.

Haematologic values	Normal	Vehicle	P100 + Dox	P200 + Dox	Dox
White blood cells (×10^3^/µL)	2.63 ± 0.10^d,e^	3.04 ± 0.22^d,e^	2.86 ± 0.24^d,e^	3.64 ± 0.04^a,b,c,e^	1.88 ± 0.27^a,b,c,d^
Neutrophil (%)	26.80 ± 3.48^b,c,d,e^	38.80 ± 2.91^a,c,d,e^	52.80 ± 2.94^a,b^	52.40 ± 4.01^a,b^	58.20 ± 2.58^a,b^
Lymphocyte (%)	60.40 ± 7.83^c,d,e^	55.33 ± 2.89^c,e^	40.60 ± 3.30^a,b^	42.80 ± 4.28^a^	28.80 ± 1.85^a,b^
Monocyte (%)	1.80 ± 0.20^e^	0.83 ± 0.17^e^	1.67 ± 0.21^e^	1.17 ± 0.17^e^	4.40 ± 1.03^a,b,c,d^
Eosinophil (%)	1.20 ± 0.37^b^	2.40 ± 0.40^c,d,e^	0.63 ± 0.32^b^	1.00 ± 0.24^b^	0.60 ± 0.40^b^
Basophil (%)	0.20 ± 0.20^e^	2.80 ± 0.86	3.00 ± 0.45	3.60 ± 0.24	4.60 ± 2.40^a^
Red blood cells (×10^6^/µL)	7.53 ± 0.20^c,d,e^	7.07 ± 0.17^c,d,e^	5.93 ± 0.30^a,b,e^	5.67 ± 0.26^a,b,e^	4.59 ± 0.61^a,b,c,d^
Platelet (×10^5^/µL)	6.67 ± 0.28^c,d^	7.18 ± 0.48^c,d^	11.10 ± 0.30^a,b,e^	10.67 ± 0.84^a,b,e^	6.35 ± 1.04^c,d^

Values represented the mean ± SEM. *p*< 0.05 significantly different compared with the a = vs. normal, b = vs. vehicle, c = vs. P100 + Dox, d = vs. P200 + Dox and e = vs. Dox.

### PFPE effect on type 1 helper T cells and suppressed type 2 helper T cells during doxorubicin treatment

**Figure 4.**
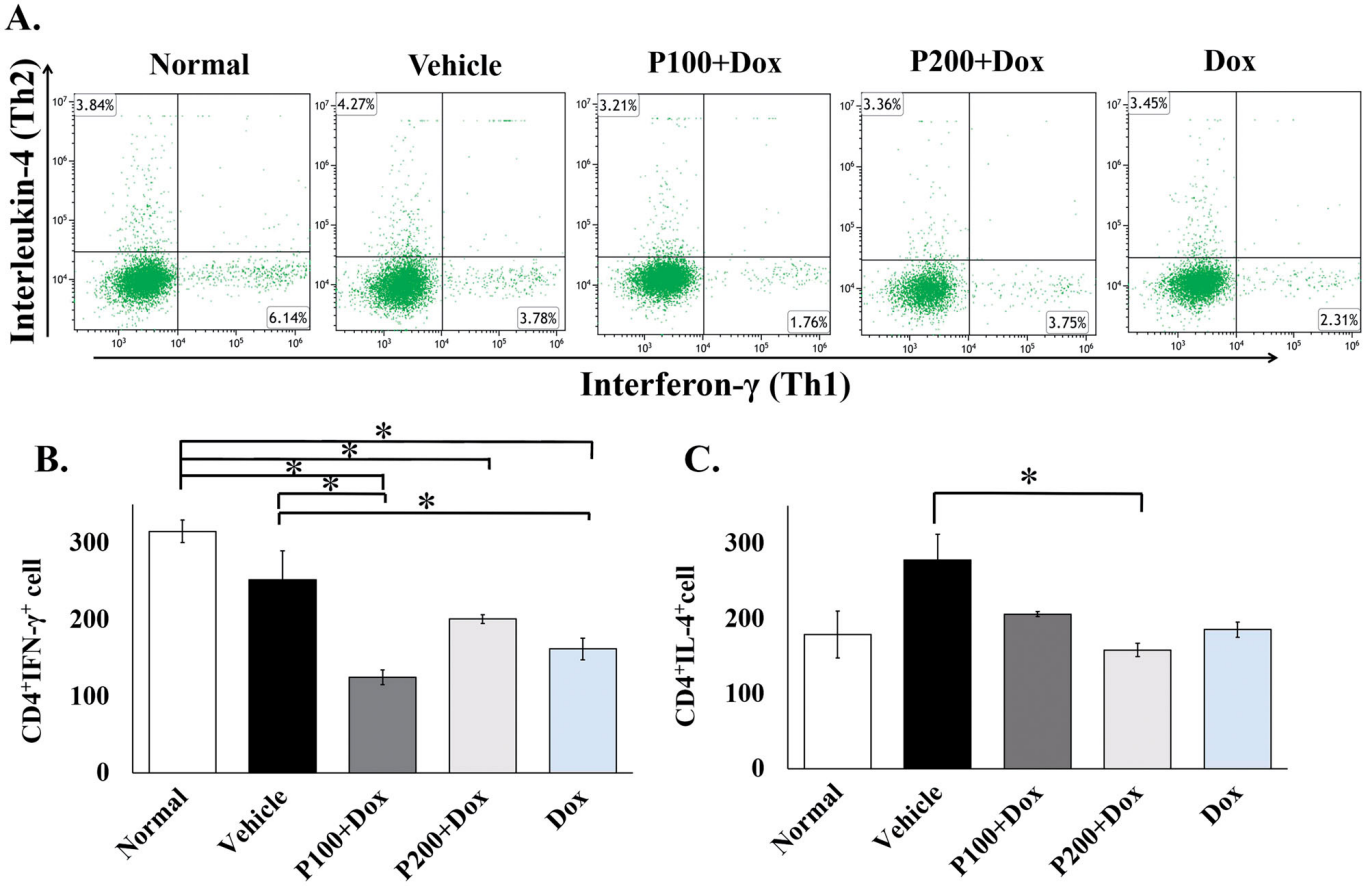
The analysis of circulating type 1 and type 2 helper T cells using flow cytometry analysis. The blood from each group of rats was collected at sacrificed day and mononuclear cells were isolated. The antibodies were used to identify both types of cells and represented as representative dot plots for Th1 (CD4^+^IFN-γ^+^) and Th2 (CD4^+^IL-4^+^) proportions (A). The number of helper T cells was calculated and represented for Th1 (B) and Th2 (C) in bar graphs (*n* = 5). Data represent the mean ± SEM (*n* = 5). *p < 0.05, compared as indicated using one-way ANOVA.

There is a great amount of evidence that demonstrates the role of the immune response to combat cancer formation and progression in the human body (Teng et al. 2015). However, the use of a chemotherapeutic drug can change the number of some kinds of leukocytes which affect antitumor immunity, especially lymphocytes (Wijayahadi et al. 2007). For this reason, we evaluated the ability of PFPE on immune protection during the Dox regimen in mammary tumour rats. The changes of helper T lymphocyte subtypes, type 1 T helper cells (Th1; CD4^+^IFN-γ^+^) and type 2 T helper cells (Th2; CD4^+^IL-4^+^), which related to anticancer immunity, were then determined by flow cytometry on sacrifice day. We found that during cancer progression the number of Th1 and Th2 cells was decreased and increased, respectively, in vehicle treatment compared to normal rats. However, Dox treatment alone decreased the levels of circulating Th1 cells around 1.95 times (*p* = 0.002) and 1.56 times (*p* = 0.057) compared to the normal and vehicle groups. Interestingly, Dox combination with 200 mg/kg BW of PFPE increased the levels of Th1 cells but did not restore Th1 cells to normal levels (Figure 4(A,B)). Furthermore, Th2 cells decreased in the treatment of Dox alone compared to the vehicle group. The combination of Dox with 200 mg/kg BW of PFPE slightly decreased the Th2 cell level when compared to Dox, single treatment (Figure 4(A,C)).

### PFPE suppressed doxorubicin induced CD4^+^CD25^+^ regulatory T cells promotion

**Figure 5.**
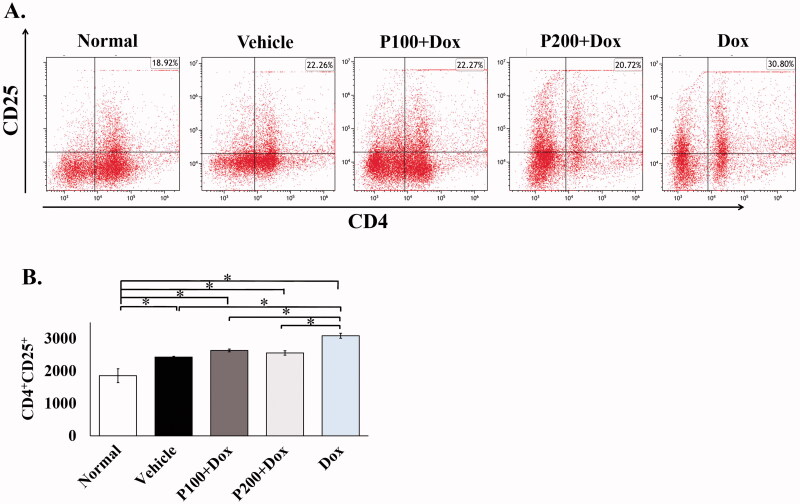
The analysis of circulating regulatory T cells using flow cytometry. The blood from each group of rats was collected at sacrificed day and mononuclear cells were isolated. The specific antibodies were used to identify the Treg cells and represented as dot plots of CD4^+^IL-25^+^ T cells (A). The number of CD4^+^IL-25^+^ cells per 10,000 mononuclear cells from representative rats (*n* = 5) was calculated and represents as mean ± SEM (B). *p < 0.05, compared as indicated using one-way ANOVA.

In addition to Th1 and Th2 cells, another type of lymphocyte that influences the antitumor immune response is the regulatory T cells (Treg; CD4^+^CD25^+^ T lymphocyte). These cells are a subset of T cells that negatively regulate immune response (Ohue and Nishikawa 2019). The high levels of Treg often associated with tumour progression and immune suppression are mediated by several mechanisms (Ohue and Nishikawa 2019). Therefore, circulating Treg from each group of rats was then evaluated by flow cytometry using CD4 and CD25 as the markers. Our study revealed that tumour bearing rats treated with Dox alone showed the highest levels of CD4^+^CD25^+^ Treg among other groups while this cell type was found with a lower number, around 1.6-fold in normal rats compared to the Dox group (Figure 5(A,B)). Interestingly, Dox combination with 100 and 200 mg/kg BW of PFPE treatment significantly reduced the levels of Treg in rats receiving Dox, approximately 1.1–1.2-fold compared to the Dox group. However, it was still higher than normal rats (Figure 5). These data indicate that Dox promoted the polarization of CD4^+^CD25^+^ Treg; while feeding rats PFPE could suppress this promotion, it could not restore it to the normal range.

## Discussion

In breast cancer, Dox is used as a drug of choice for patients who are unresponsive to other regimens or metastasis (Gariboldi et al. 2003). However, behind the effective therapy response, this chemotherapeutic agent also provides unfavourable outcomes, such as leukocyte depletion and bone marrow damage (Nurgalieva et al. 2011), hematotoxicity, hair loss (Baltali et al. 1999), irreversible cardiomyopathy (Chatterjee et al. 2010) and bone loss (Rana et al. 2013). Various studies have reported that all these complications are often associated with the pro-inflammatory properties of Dox that induce inflammation in many sites of the body, including the liver, kidney, intestines and blood vessels (Kaczmarek et al. 2012; Chen et al. 2013; Leal et al. 2014; Wang et al. 2016). The mechanism of inflammation causes by Dox is associated with the activation of NFκB, COX-2 and inducible nitric oxide synthase (iNOS) (Dowd et al. 2001; Octavia et al. 2012; Rehman et al. 2014; Yang et al. 2020). Nowadays, some anti-inflammation compounds have been found to alleviate these Dox-induced adverse events (Benzer et al. 2018; Guo et al. 2020; Jiang and Zhang 2020). *P. nigrum* is one of the medicinal plants recognized to contain anti-inflammation properties (Butt et al. 2013). GC–MS analysis of our *P. nigrum* extract, PFPE, revealed many substances that were found to inhibit inflammatory mediators, and some compounds have been found to reduce the toxicity of Dox directly. For example, caryophyllene, the major compound of PFPE, has been demonstrated to display the reduction effect on prostaglandin E(2) (PGE(2)), as well as iNOS and cyclooxygenase (COX-2) expression (Fernandes et al. 2007), which lead to the attenuation of oxidative stress and inflammation caused by Dox in rats (Al-Taee et al. 2019). Limonene is another compound found in PFPE that reduced Dox induced kidney injury through the suppression of COX-2, iNOS and NFκB (Rehman et al. 2014). Other substances, such as pellitorine, β-selinene, γ-terpinene or whole black pepper extract, have also displayed anti-inflammation properties (Lee et al. 2014; Ramalho et al. 2015; Bui et al. 2017; Chandra et al. 2017). This suggests that PFPE may help to reduce the cytotoxic effect and pro-inflammatory response of Dox through anti-oxidative and anti-inflammatory capabilities.

Our study demonstrated that the combination of PFPE and Dox did not show any disruptive effect on tumour inhibitory efficacy. However, Dox treatment alone may play a role in organ toxicity since the higher organ/body weight ratio of some sites was found, but the PFPE combination could lower this complication, emphasizing the role of PFPE in attenuating Dox toxicity. The protective role of PFPE on internal organ damage is also supported by the activity of the haematologic chemical parameter, AST, that increased when the injury of some organs, such as cardiac muscle, skeletal muscle, kidneys, brain, pancreas, lungs, leucocytes and erythrocytes, was found (Fellowship Examination of the Royal College of Pathologists (FRCPath) 2017). However, when both doses of PFPE were combined in mammary tumour treatment, AST levels were decreased, indicating the cytoprotective role of PFPE.

Doxorubicin also influenced the nutritional status of the rats. The parameters related to protein metabolisms, such as BUN, creatinine, albumin and alkaline phosphatase, showed lower levels than normal and vehicle rats suggested the role of Dox in anorexia, which led to diminished protein intake and malnutrition (Wallig et al. 2017). Moreover, Dox treatment also caused blood dyslipidemia with the increase of cholesterol and triglyceride levels that could be related to the reduction of lipolysis rate (Hong et al. 2002; Chennuru and Saleem 2013). However, PFPE could restore some parameters near to the normal range. Importantly, the PFPE sesquiterpene substance, β-caryophyllene, has been reported to effectively act as an anti-hyperlipidemia agent, same as the reference drug simvastatin, through the suppression of HMG-CoA reductase, which is the enzyme that plays a role in cholesterol synthesis (Baldissera et al. 2017).

The immunological and haematological effects of Dox were also demonstrated by our study. The suppression of red blood cells was observed in the Dox group, and may be caused by haemoglobin oxidation and an increase in erythrocyte fragility (Shinohara and Tanaka 1980; Oto et al. 2015; Sergazy et al. 2020). Importantly, PFPE feeding improved the levels of red blood cells, which may be due to the antioxidant properties of PFPE. This is in accordance with other studies that demonstrated the use of antioxidant substances, such as polyphenol, could protect this erythrocyte damage from Dox (Sergazy et al. 2020). Unexpectedly, while using Dox alone did not change platelet levels when compared to normal rats, the combination of Dox and PFPE increased the levels of the platelets. Although the mechanism of this scenario has not been fully elucidated, there is evidence showing some plant extract, *Carica papaya* L. (Caricaceae) leaf extracts, increased the number of platelets in animals and humans (Dharmarathna et al. 2013; Gadhwal et al. 2016). Interestingly, the analysis of chemical compounds found in *C. papaya* extract included alkaloids, tannins, anthraquinone, cardenolides, steroids, saponins, etc. (Dharmarathna et al. 2013), some of which related to compounds in PFPE.

The decreased levels of total white blood cells and lymphocytes were revealed as expected. This scenario can be explained by other studies showing that Dox treatment led to the depletion of lymphocytes in peripheral lymphoid organs (Pourtier-Manzanedo et al. 1995), lymphocyte stem cells dysfunction and mature lymphocyte destruction (Steele 2002). Importantly, the role of antioxidants has been reported in restoring these complications (Merzoug et al. 2014), which may support the role of substances in PFEP in preventing lymphocyte damage. However, Dox treatment resulted in a high number of neutrophils and monocytes. These different effects on different types of immune cells may be from the specificity of the action of Dox that targets and damages rapidly proliferative cells, such as lymphocytes (Douedi and Carson 2020).

Circulating cytokines can be used as a marker to monitor the immune status of the body. In the aspect of tumour immunobiology, tumour microenvironment has been proposed for its role in cancer progression and metastasis, and this scenario requires interaction between cancer cells, immune cells and tumour stroma, which consequently reflect the immune response of the patients (Hirata and Sahai 2017). Cytokine profiling of rats treated with different types of regimens demonstrated the role of Dox and PFPE-Dox combination on the immune response. Interestingly, the levels of CXCL-7, TIMP-1, sICAM-1 and l-selectin of the vehicle and Dox groups seemed to be equal, although Dox inhibited tumour progression. In addition, all of those cytokines have been reported for their role in the tumour promoting function (Yamada et al. 2005; Ridnour et al. 2012; Grepin et al. 2014). This phenomenon may indicate the immunological response which still mimicked the tumour promoting environment influenced by Dox despite the suppression of the tumour being found. It was noticeable that the use of PFPE suppressed all of these cytokines in the Dox group, and some compounds in PFPE were remarkably demonstrated to inhibit these cytokines directly in the tumour model. To illustrate, 3-carene and pellitorine were found to inhibit the expression of CXCL7 through the suppression of IL-6 from cancer cells leading to cancer elimination (Chiang et al. 2012; Ku et al. 2014; Basholli-Salihu et al. 2017). Caryophyllene and α-humulene were also reported to suppress sICAM-1 production (Tanaka et al. 2001). Moreover, TIMP-1 expression could be inhibited by β-caryophyllene treatment in the liver fibrosis animal model (Calleja et al. 2013). This demonstrated that all of these antioxidants in PFEP could synergistically help to suppress a tumour-promoting immunological environment.

In addition to cytokine profiling, we also examined the effect of Dox and PFPE-Dox combination on antitumor related immune cells. Our previous work showed that PFPE promoted the antitumor immune response by regulating Th1, Th2 and Treg cells (Saetang et al. 2021). Therefore, all types of these cells were included in this study. It has been shown that the tendency of pro-tumorigenic helper T cells (Th2) and antitumorigenic helper T cells (Th1) were increased and decreased in vehicle, respectively, while Dox treatment suppressed both Th1 and Th2. This emphasized our hypothesis describing the role of Dox in lymphocyte depletion. However, the addition of 200 mg/kg BW of PFPE in the Dox group seemed to promote the number of Th1 and obviously reduced the levels of Treg, indicating the role of PFPE in immunomodulation. Moreover, other studies also supported the promoting role of *P. nigrum* extract on the polarization of Th1 immunity (Majdalawieh and Carr 2010; Bui et al. 2017). Specifically, it has been reported that a compound in PFPE, α-humulene, recovered IFN-γ production leading to the activation of Th1 immune response in the animal model (Rogerio et al. 2009). Doxorubicin also promoted a pro-tumorigenic environment, reflected by the increasing number of Treg cells which play a role in immunosuppression (O'Donnell et al. 2019). This could be explained by the pro-inflammatory response induced by Dox, which promoted the high levels of neutrophils and monocytes leading to the pro-inflammatory cytokine production and oxidative stress response (Bhagat et al. 2020). This scenario caused the polarization of Treg cells to control the inflammation and tissue injury (Lei et al. 2015). Interestingly, PFPE decreased the levels of this type of cell, which may be because of the antioxidant and anti-inflammation components constituted in this extract, leading to the suppression of Dox-induced inflammatory response.

Taken together, this is the first study of the role of PFPE in attenuating Dox-induced toxicity in the mammary tumour model. The antioxidant and anti-inflammation properties of PFPE may play a role in this phenomenon. Moreover, PFPE also displayed antitumor promoting properties in the situation of Dox treatment by induction of Th1 cells. This emphasized the immunomodulation effects that might be used to minimize the toxicity of chemotherapeutic drugs on the immunity of cancer patients. Although this investigation revealed the role of PFPE in Dox toxicity reduction, the protective role of PFPE on the main side effects of Dox, such as cardiomyopathy, should be investigated. Furthermore, the other types of T cells, including Th17, Th9, Th22 and other immune cells, should also be examined in the future.

## Conclusions

In our previous work, we demonstrated the role of PFPE in promoting antitumor immunity. However, the effect of this extract when combined with chemotherapeutic drugs has not been reported. This is the first study that has revealed the potential of PFPE to be used as an additive for Dox treatment. While the use of PFPE did not disrupt the anticancer property of Dox, it alleviated the toxicity of this drug in many aspects. Rats treated with Dox alone showed a higher organ/body weight ratio, but combining Dox with PFPE could lower the ratio in all the organs, except the kidneys. Doxorubicin also influenced white blood cell count, many blood parameters, and some were related to protein metabolism and organ damage. The combination of either 100 or 200 mg/kg of PFPE could restore all blood chemistry to near normal levels and attenuate the lymphocyte suppressing activity of Dox. PFPE also decreased protumorigenic cytokine induced by Dox, and increased the number of Th1 cells, meanwhile, both doses of PFPE suppressed the number of Treg close to normal levels. Taken together, these data indicate that PFPE can be used in combination with Dox to reduce its toxicity.

## Supplementary Material

Supplemental Material
