# Supplementary material for: The attenuation effect of low piperine Piper nigrum extract on doxorubicin-induced toxicity of blood chemical and immunological properties in mammary tumour rats
Source: Pharm Biol. 2021 Dec 28;60(1):96–107. doi: 10.1080/13880209.2021.2018470 (PMC8735876; doi:10.1080/13880209.2021.2018470)
Supplement: Supplemental Material [file IPHB_A_2018470_SM7374.docx]

**
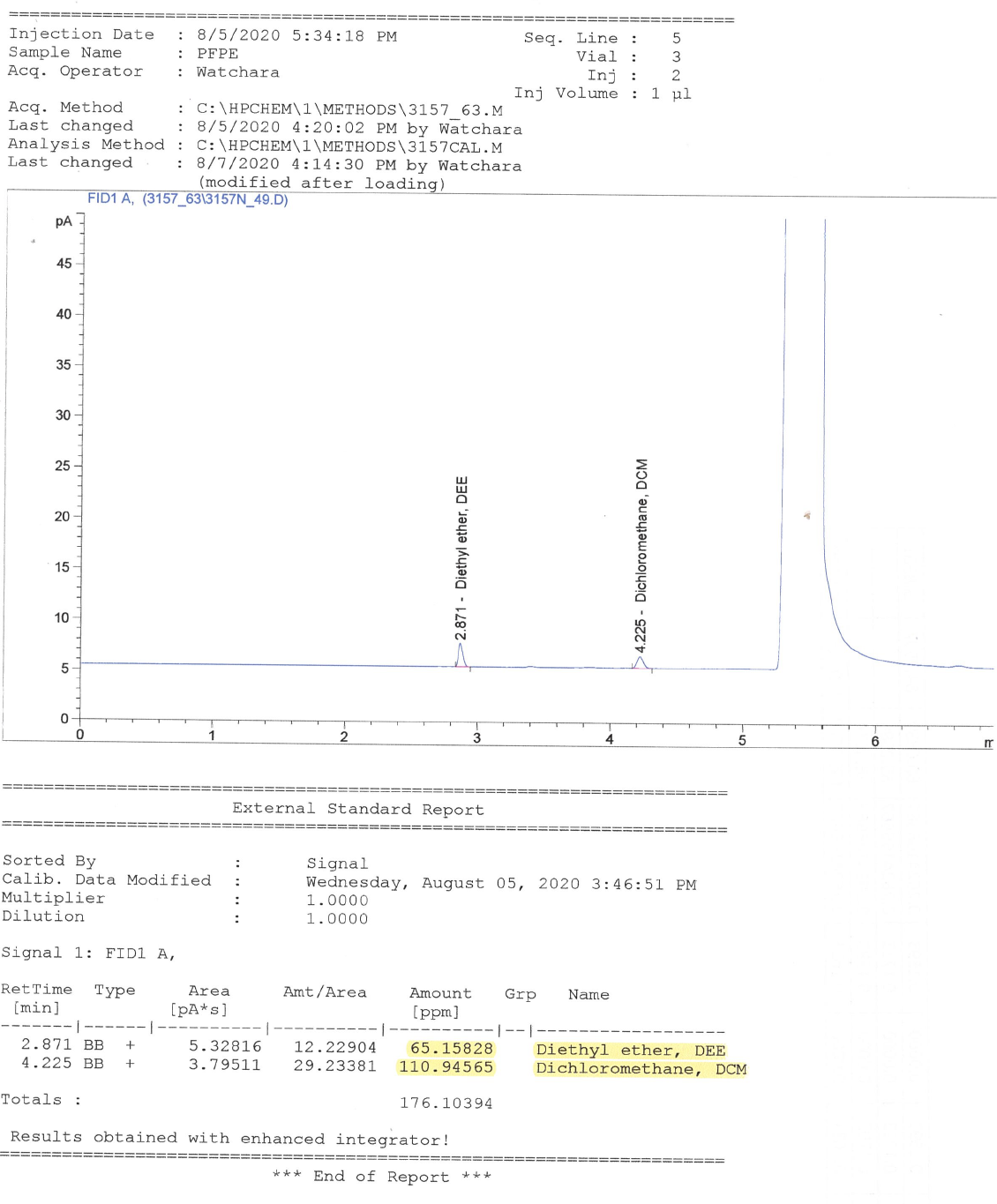
Figure S1.** The representative of chromatogram analysis of PFPE from GC-FID showing the levels of dichloromethane and diethyl ether in the extract.

**Figure S2.** The representative result of MTT analysis of PFPE on MCF-7 cell line.
